# Cardiorespiratory fitness attenuates the association between psychosocial stress and cardiometabolic risk – Results from the SCAPIS population

**DOI:** 10.1371/journal.pone.0345029

**Published:** 2026-03-16

**Authors:** Frida Griffin, Jonatan Fridolfsson, Daniel Arvidsson, Ingibjörg H. Jonsdottir, Mats Börjesson

**Affiliations:** 1 Department of Molecular and Clinical Medicine, Institute of Medicine, Sahlgrenska Academy, University of Gothenburg, Gothenburg, Sweden; 2 Center for Lifestyle Intervention, Sahlgrenska University Hospital, Region Västra Götaland, Gothenburg, Sweden; 3 Center for Health and Performance, Department of Food and Nutrition and Sport Science, Faculty of Education, University of Gothenburg, Gothenburg, Sweden; 4 School of Public Health and Community Medicine, Institute of Medicine, Sahlgrenska Academy, University of Gothenburg, Gothenburg, Sweden; 5 Institute of Stress Medicine, Region Västra Götaland, Gothenburg, Sweden; Kurume University School of Medicine, JAPAN

## Abstract

**Aims:**

Low cardiorespiratory fitness (CRF) and high psychosocial stress can contribute to cardiovascular disease. CRF is a strong predictor of cardiovascular outcomes, yet population-based evidence on whether CRF buffers stress-related risk remains limited. This study aims to (1) examine associations between CRF, stress, and cardiometabolic risk; (2) assess whether CRF moderates the relationship between stress and cardiometabolic risk; and (3) explore whether associations between CRF and cardiometabolic risk are stronger among high-stress individuals.

**Methods:**

We included 4,207 healthy, middle-aged participants from the Swedish CArdioPulmonary bioImage Study (SCAPIS), conducted 2013–2018. CRF was estimated using submaximal cycle testing (ml/min/kg). Perceived psychosocial stress was measured using a single self-reported item dichotomised into “low” and “high”. Ten cardiometabolic outcomes were assessed, including waist circumference, BMI, and blood pressure. Cross-sectional associations were analysed in R, using t-tests and multiple linear regression.

**Results:**

Individuals reporting high stress had lower CRF (−1.7 ml/min/kg, p < .001) and less favourable cardiometabolic profiles. CRF moderated the relationship between stress and waist circumference, BMI, systolic- and diastolic blood pressure. For each 1 ml/min/kg higher CRF, the stress-related association was reduced by 0.17 cm in waist circumference (p < .001), 0.06 kg/m^2^ in BMI (p < .001), 0.18 mmHg in systolic blood pressure (p = .030), and 0.13 mmHg in diastolic blood pressure (p = .020). Associations were 13–25% stronger in the high-stress group.

**Conclusion:**

Higher CRF attenuated the association between psychosocial stress and cardiometabolic risk. Promoting physical activity to improve CRF could be important during periods of high stress to counteract stress-related cardiometabolic deterioration.

## Introduction

Cardiovascular disease remains the leading cause of morbidity and mortality worldwide, with physical inactivity and psychosocial stress recognised as major modifiable risk factors [1]. In the adult Swedish population, two alarming public health trends have emerged in recent decades: a steady decline in cardiorespiratory fitness (CRF) to notably low levels [2], alongside significantly deteriorating perceived general health [3]. In addition, stress-related psychiatric diagnoses have become the leading causes of long-term sick leave in the Swedish working-age population, with highest prevalence observed in middle-aged and older adults [4,5]. Both low CRF and high psychosocial stress increase cardiovascular disease risk [6–10], yet their co-occurrence and potential interactions remain poorly understood.

CRF has emerged as a particularly strong predictor of cardiovascular disease [9,11–15], potentially surpassing traditional risk factors such as smoking, hypertension, and type 2 diabetes in predicting all-cause mortality [16]. Follow-up studies demonstrate that improvements in CRF correlate with better cardiovascular risk profiles [17–19], reduced hospitalisation rates [20], and lower overall mortality [21–24].

High levels of psychosocial stress are similarly linked to higher cardiometabolic risk, including elevated low-density lipoprotein cholesterol, triglycerides, and total metabolic risk [7], as well as a greater incidence of cardiovascular events [25] and premature death [26,27]. Psychosocial stress affects multiple psychobiological pathways, including the sympathetic nervous system and hypothalamic–pituitary–adrenal axis, affecting blood pressure, insulin sensitivity, inflammatory responses, and endothelial function [6,28]. Chronic psychosocial stress may also foster harmful lifestyle behaviours such as physical inactivity, smoking, and poor dietary habits [29–31], further amplifying these biological processes [6].

Physical activity can counteract plausible biological consequences of chronic psychosocial stress, possibly affecting autonomic balance, improving endothelial function, lowering systemic inflammation and improving metabolic regulation [6,32,33]. Thus, physical activity has been proposed as a potential buffer against stress-related cardiovascular effects [6,33]. Physical activity and CRF represent related but distinct concepts. Physical activity refers to any bodily movement that increases energy expenditure, whereas CRF reflects the body’s physiological capacity to deliver and utilise oxygen during sustained activity [34].

While several studies have shown relationships between exercise behaviour assessed using self-reporting, and attenuation of the impact of psychosocial stress [27,35,36], self-reported measures are prone to recall and social desirability biases, correlate weakly with objective measures, and show weaker associations with morbidity and mortality outcomes [37–39]. By contrast, CRF captures habitual physical activity, as well as individual physiological responses, making it a robust and clinically relevant marker of long-term cardiovascular adaptation beyond what self-reported exercise alone can detect [40–42].

Despite this potential, evidence examining objectively measured CRF as a moderator of stress-related cardiometabolic outcomes remains limited and shows mixed findings depending on the stress paradigm studied. Most research has utilised laboratory-based acute psychological stressors rather than real-world, chronic psychosocial stress. For example, a 2006 meta-analysis of 33 studies examining acute psychological stressors found that individuals with higher CRF exhibited significantly attenuated heart rate and systolic blood pressure reactivity in controlled laboratory settings [32]. However, these findings were derived from short-duration, experimentally induced stress exposures that may not translate directly to chronic psychosocial stressors encountered in daily life. Evidence for CRF buffering such chronic real-world stress is scarce and inconsistent. A 2009 review concluded that while higher exercise levels were associated with fewer health-related complaints, evidence for CRF protecting against cardiometabolic consequences of chronic psychosocial stress remained limited [36].

More recently, Gerber et al. [7] reported that among individuals experiencing high chronic stress, those with high CRF demonstrated more favourable cardiometabolic profiles than their low-CRF counterparts, including lower blood pressure, low-density lipoprotein cholesterol, triglycerides, and total metabolic risk. However, that study was limited by its small, selective sample (N = 197) of predominantly healthcare professionals aged 25–50 years, restricting generalisability to broader populations.

Given concurrent declines in CRF and high stress-related morbidity, understanding whether CRF mitigates psychosocial stress-related cardiometabolic risk at the population level has important public health implications. If CRF buffers psychosocial stress effects, promoting CRF could simultaneously address the direct consequences of physical inactivity and indirect effects of psychosocial stress. Large population-based studies are needed to examine this relationship across the full spectrum of CRF levels, psychosocial stress exposure, and cardiometabolic risk profiles.

The present study therefore investigates the relationship between CRF, psychosocial stress, and cardiometabolic risk in a large population-based sample. Specifically, we aim to (1) examine associations between CRF, psychosocial stress, and cardiometabolic risk; (2) assess whether CRF moderates the relationship between psychosocial stress and cardiometabolic risk; and (3) explore whether associations between CRF and cardiometabolic risk are particularly pronounced among individuals experiencing high psychosocial stress.

## Materials and methods

### Study sample

This population-based cohort study included 4,207 middle-aged women and men from the Gothenburg site of the Swedish CArdioPulmonary bioImage Study (SCAPIS). SCAPIS comprises approximately 30,000 randomly selected adults aged 50–64 years (51% women), conducted between 2013 and 2018 across six Swedish sites: Gothenburg, Linköping, Malmö/Lund, Stockholm, Umeå, and Uppsala [43].

The six sites were selected to represent Sweden’s geographic and demographic diversity. The distribution was intended to capture variation in urbanisation levels, economic profiles, and population characteristics. Although comparative data across SCAPIS sites have not been published, the uniform recruitment approach across centres was designed to support population representativeness at the national level.

The SCAPIS protocol included comprehensive questionnaires, biochemical analyses, anthropometric measurements, and cardiovascular imaging, among other assessments. Additionally, submaximal CRF testing was conducted at the Gothenburg site using a bicycle ergometer. Therefore, in this study, only individuals from the Gothenburg site were included.

Of the 6,265 participants initially enrolled at the Gothenburg site, Sweden’s second-largest city, we identified those with valid data for CRF (n = 4,513), self-perceived psychosocial stress (n = 6,172), and cardiometabolic outcomes (n = 5,853). Participants did not undergo CRF testing if they had ongoing infections, unstable cardiovascular disease, electrocardiographic patterns indicating cardiac disease, were using beta-blockers, had a resting heart rate above 100 beats per minute, or weighed more than 125 kilograms. The primary reason for missing data was failure to meet test criteria or opting out of CRF testing (n = 1,752 of the 6,265 participants at the Gothenburg site). For the current analyses, we only included participants with complete data for all variables of interest, including age and sex (n = 4,207). Participants who completed CRF testing were younger on average, had lower BMI, were more likely to engage in exercise, and had higher educational levels [44].

The SCAPIS study was approved by the Ethics Review Board in Umeå (approval number 2010–228-31M). All participants provided written informed consent, and the current study was further approved by the Regional Ethics Board in Gothenburg (approval number 638–16, including approved extension 2020–02606). Data was accessed 24^th^ of August 2023 in an anonymised format and no individual participants could be identified at any point.

### Data collection and variables

#### Cardiorespiratory fitness.

CRF reflects maximal oxygen consumption (VO_2_max) and represents the integrated capacity of the cardiovascular, pulmonary, and muscular systems to deliver and utilise oxygen during sustained physical activity [34]. In SCAPIS, CRF was estimated using the Ekblom–Bak two-point submaximal exercise test on a calibrated cycle ergometer and was expressed in ml/min/kg [45]. In the hours preceding the test, participants were instructed to avoid drinking coffee or consuming heavy meals. Each participant cycled at two work rates for four minutes each, maintaining a pedal frequency of 60 revolutions per minute: first, a lower standard rate of approximately 30 watts; and a second, higher individually adjusted rate corresponding to moderate intensity. At each rate, mean heart rates were recorded during the final minute of cycling. Values from the test were subsequently entered into sex-specific validated algorithms, together with age, for VO_2_max estimation. The Ekblom–Bak test has been validated against directly measured VO_2_max in similar age groups, and has demonstrated small, non-significant systematic error [45].

#### Psychosocial stress.

Psychosocial stress was self-reported using a single-item question in the SCAPIS questionnaire. The item incorporates self-perceived stress at work and at home and reads; “*By stress we mean feeling tense, irritable, anxious or having sleeping difficulties as a result of conditions at work or at home. Did you experience this?”* Participants responded using a five-point Likert scale: (1) “never”, (2) “some periods”, (3) “several periods during the last five years”, (4) “permanent stress during the last year”, and (5) “permanent stress during the last five years”. For the present analyses, perceived psychosocial stress was dichotomised into “low” (responses 1–3) and “high” (responses 4–5), in line with previous studies [25,46]. For readability, we refer to perceived psychosocial stress as “stress” throughout this manuscript.

#### Cardiometabolic risk markers.

Ten cardiometabolic risk markers were assessed: waist circumference, body mass index (BMI), systolic blood pressure (SBP), diastolic blood pressure (DBP), low-density lipoprotein cholesterol (LDL-C), high-density lipoprotein cholesterol (HDL-C), triglycerides (Tg), glycated haemoglobin (HbA1c), glucose, and the Systematic Coronary Risk Evaluation-1 (SCORE-1) system.

Waist circumference (cm) was measured using a non-stretchable tape at the midpoint between the top of the iliac crest and the lower rib margin, following exhalation. Weight was measured in light clothing and rounded to the nearest 0.1 kilograms (kg) using a balance scale, and height was measured using standard techniques and rounded to the nearest 0.1 cm. BMI was calculated as weight (kg) divided by height squared (m^2^).

Blood pressure (mmHg) was measured twice in both arms using an automated device (Omron M10-IT, Omrom Healthcare Co, Kyoto, Japan) after five minutes of rest. The average of the two readings from the arm with the higher mean value was used. Venous blood samples were collected following an overnight fast. HbA1c (mmol/mol), glucose, HDL-C, and Tg (mmol/L) were measured by standard techniques. LDL-C was calculated according to Friedewald’s formula: LDL-C = [Total cholesterol – HDL-C – (0.45 x Tg)].

SCORE-1 is a risk factor burden model which estimates the 10-year risk of fatal atherosclerotic cardiovascular disease and is expressed as a percentage value [47]. A SCORE value <2% is generally considered as low risk, 2% to 5% as moderate risk, and >5% as high 10-year risk of fatal cardiovascular disease. SCORE-1 is calculated based on age, sex, SBP, cholesterol levels, and smoking status (self-reported). SCORE-1 is referred to as “SCORE” throughout this manuscript.

#### Other variables.

Age and sex were obtained from national registers and reported in SCAPIS. For descriptive tables, self-reported educational level and marital status were included. Educational level was categorised as “low” (≤9 years of primary/lower secondary education or upper secondary education) and “high” (university or college degree). Marital status was classified as “not married” (single, divorced, or widowed) or “married”.

### Statistical analyses

Descriptive statistics are presented as means ± standard deviations (SD) for continuous variables and as counts and percentages for categorical variables. Independent samples *t*-tests were used to compare CRF levels between low- and high-stress groups in the total sample, and separately for women and men.

Multiple linear regression analyses were performed to examine associations of CRF, stress (low stress as reference), and their interaction (CRF x stress) with each cardiometabolic risk marker. Prior to running the regressions, standard assumptions were checked, and no violations were detected. Each outcome (waist circumference, BMI, SBP, DBP, HbA1c, glucose, HDL-C, LDL-C, Tg, and SCORE) was analysed in a separate model, with age and sex included as covariates.

Age and sex are biological determinants that directly influence CRF and cardiometabolic risk and would therefore be considered as confounders. In contrast, educational level and marital status are socioeconomic preconditions influencing behaviours (e.g., physical activity), which in turn influence CRF, stress, and cardiometabolic risk. Including variables in the multiple regression that are in a potential causal pathway, interferes with their true associations and introduces analytical errors. Therefore, educational level and marital status were included for descriptive purposes only.

To facilitate interpretation of interaction effects, all continuous predictors were mean-centred prior to inclusion in the regression models. In this way, the main effects represent the association at average levels of the interacting variable (i.e., average CRF), and the interaction term captures the differential effect of CRF at higher versus lower levels of stress. In addition, all continuous variables were standardised using z-transformation (mean = 0, SD = 1), to allow for comparison of relative effect sizes across outcomes. Both centred and standardised models are reported.

Statistical significance was set at p < .05. Adjusted R-squared values were used to assess the proportion of explained variance in each regression model. All analyses were conducted in R (version 4.4.2; R Core Team, 2024, R Foundation for Statistical Computing, Vienna, Austria), using the Tidyverse, readxl, writeexl, and flextable packages.

## Results

### Descriptive characteristics

A total of 4,207 participants (51% women) were included in the analyses. Of these, 78% (n = 3,288) reported low levels of stress, while 22% (n = 919) reported high stress. Descriptive characteristics of the study sample are presented in Table 1.

**Table 1:** Descriptive characteristics of the study sample, presented as means (SD), counts, and percentages.

		Total (N = 4,207)	Low stress (N = 3,288)	High stress (N = 919)
Sex	Women	2147 (51.0%)	1561 (47.5%)	586 (63.8%)
	Men	2060 (49.0%)	1727 (52.5%)	333 (36.2%)
Age (years)		57.2 (4.3)	57.3 (4.3)	56.6 (4.1)
†Educational level	Low	2164 (51.4%)	1740 (52.9%)	424 (46.1%)
	High	2043 (48.6%)	1548 (47.1%)	495 (53.9%)
‡Marital status	Not married	1221 (29.0%)	898 (27.3%)	323 (35.1%)
	Married	2986 (71.0%)	2390 (72.7)	596 (64.9%)
CRF (ml/min/kg)		33.9 (6.7)	34.2 (6.7)	32.6 (6.6)
Waist circumference (cm)		92.5 (12.0)	92.5 (11.9)	92.7 (12.3)
BMI (kg/m^2^)		26.4 (4.0)	26.3 (3.9)	26.8 (4.3)
Systolic BP (mmHg)		121.3 (16.3)	121.7 (16.4)	119.8 (15.9)
Diastolic BP (mmHg)		72.5 (10.2)	72.5 (10.2)	72.3 (10.1)
LDL-C (mmol/L)		3.7 (0.9)	3.7 (0.9)	3.7 (0.9)
HDL-C (mmol/L)		1.7 (0.5)	1.7 (0.5)	1.7 (0.5)
Triglycerides (mmol/L)		1.2 (1.0)	1.2 (1.0)	1.2 (0.8)
HbA1c (mmol/mol)		35.0 (5.0)	34.9 (5.0)	35.4 (5.1)
Glucose (mmol/L)		5.6 (0.9)	5.6 (0.9)	5.7 (1.0)
SCORE (%)		1.3 (1.2)	1.3 (1.3)	1.0 (0.9)

†Educational level is dichotomised as “low” (primary/lower secondary education ≤9 years or upper secondary education) and “high” (university or college degree).

‡Marital status is dichotomised as “not married” (single, divorced, or widowed) and “married”.

### Association between CRF, stress and cardiometabolic outcomes

Independent samples *t*-tests revealed a statistically significant difference in CRF between the low- and high-stress groups (Table 2). Participants in the low-stress group had a higher mean CRF (34.2 ± 6.7 ml/min/kg) compared to those in the high-stress group (32.6 ± 6.6 ml/min/kg), corresponding to a mean difference of 1.7 ml/min/kg (p < .001). When stratified by sex, a difference between the low- and high-stress groups remained significant among women (low stress: 31.3 ± 6.0; high stress: 30.4 ± 6.0; mean difference = 0.9 ml/min/kg, p = .002), but not among men (low stress: 36.9 ± 6.1; high stress: 36.4 ± 5.7; mean difference = 0.5 ml/min/kg, p = .131). Nonetheless, a non-significant trend toward higher CRF in the low-stress group was also observed among men.

**Table 2:** Differences in estimated cardiorespiratory fitness (CRF) in the total cohort and stratified by sex.

	Low stress		High stress				
	*Mean*	*SD*	*Mean*	*SD*	*Mean difference*	*N*	*p*
CRF (total)	34.2	6.7	32.6	6.6	1.7	4207	<.001
CRF (women)	31.3	6.0	30.4	6.0	0.9	2147	.002
CRF (men)	36.9	6.1	36.4	5.7	0.5	2060	.131

Results from the multiple linear regression analyses are presented in Table 3. CRF was inversely associated with all ten outcome variables, and perceived stress showed a significant association with HDL-C and HbA1c, and a borderline-significant association with glucose.

**Table 3:** Associations between cardiorespiratory fitness (CRF in ml/min/kg), perceived stress, and cardiometabolic outcomes.

	*CRF*			*Stress*			*CRF x Stress*			
	B_cent	β	p	B_cent	β	p	B_cent	β	p	R^2^
Waist	−1.27	−0.71	<.001	0.55	0.05	.057	−0.17	−0.09	<.001	0.595
BMI	−0.45	−0.76	<.001	0.08	0.02	.449	−0.06	−0.10	<.001	0.480
SBP	−0.71	−0.29	<.001	−1.10	−0.07	.055	−0.18	−0.08	.030	0.154
DBP	−0.53	−0.34	<.001	−0.28	−0.03	.435	−0.13	−0.08	.020	0.128
LDL-C	−0.02	−0.15	<.001	−0.04	−0.04	.266	0.00	0.02	.574	0.020
HDL-C	0.03	0.35	<.001	−0.06	−0.11	<.001	0.00	0.01	.655	0.296
Tg	−0.04	−0.27	<.001	0.04	0.04	.303	0.00	0.03	.421	0.086
HbA1C	−0.11	−0.14	<.001	0.42	0.08	.027	−0.05	−0.07	.053	0.037
Glucose	−0.03	−0.23	<.001	0.07	0.07	.050	−0.00	−0.05	.148	0.087
SCORE	−0.02	−0.13	<.001	−0.04	−0.04	.179	0.00	−0.00	924	0.506

*Note.* Results from multiple regression model (95% CI) for all cardiometabolic outcomes, with CRF, stress and an interaction term (CRF x stress) as predictors, while controlling for age and sex. Presented as centered unstandardised coefficients (B_cent) and standardised coefficients (β), including p-values (p).

### Moderating effects of CRF

Significant interaction effects between CRF and stress were observed for four cardiometabolic outcomes: waist circumference, BMI, SBP, and DBP (Table 3). No significant interactions were found for the remaining outcomes. In secondary analyses, we examined whether the moderating effects identified differed across sex, educational level, or marital status. No statistically significant three-way interactions were observed for waist circumference, BMI, SBP, or DBP, indicating that the magnitude of CRF’s moderating effect did not differ by sex, educational level, or marital status.

**Fig 1:**
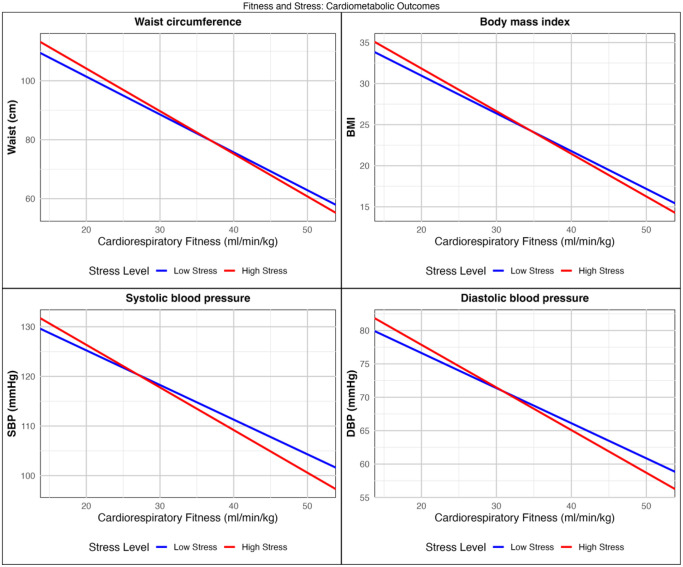
Association between cardiorespiratory fitness (CRF in ml/min/kg) and cardiometabolic outcomes, stratified by low versus high stress. The lines show predicted values from adjusted models, based on sample means for age and sex. CRF values are plotted across a range corresponding to ±3 standard deviations from the cohort mean.

The buffering effect of CRF on the association between stress and these outcomes was strongest in the high-stress group. Higher CRF was associated with more favourable cardiometabolic outcomes in both stress groups, but with steeper slopes observed in the high-stress group (Fig 1). This suggests that individuals experiencing high stress may benefit more from higher CRF, illustrating its potential buffering effect on the association between stress and cardiometabolic health.

For each 1 ml/min/kg higher CRF, waist circumference was decreased by 1.27 cm (p < .001) in the low-stress group, with an additional reduction of 0.17 cm (p < .001) in the high-stress group. This corresponds to a total reduction of 1.44 cm in the high-stress group for each 1 ml/min/kg higher CRF (combining the main effect observed in the low-stress group B_(cent)_ = −1.27 with the additional reduction captured by the interaction term B_(cent)_ = −0.17). This corresponds to an approximately 13.4% stronger association in the high-stress group, in comparison to their low-stress counterparts ([interaction effect/ main effect] x 100). The full model explained 59% of the variance in waist circumference (adjusted R^2^ = .595).

For each 1 ml/min/kg higher CRF, BMI was decreased by 0.45 kg/m^2^ (p < .001) in the low-stress group, with an additional reduction of 0.06 kg/m^2^ (p < .001) in the high-stress group. The total reduction for the high-stress group was 0.52 kg/m^2^ for each 1 ml/min/kg higher CRF (combined main effect B_(cent)_ = −0.45 and interaction effect B_(cent)_ = −0.06). This corresponds to an approximately 13.3% stronger association in the high-stress group. The full model explained 48% of the variance in BMI (adjusted R^2^ = .48).

For each1 ml/min/kg higher CRF, SBP was decreased by 0.71 mmHg (p < .001) in the low-stress group, with an additional reduction of 0.18 mmHg (p = .030) in the high-stress group, corresponding to a total reduction of 0.89 mmHg for the high-stress group, for each 1 ml/min/kg higher CRF (combined main effect B_(cent)_ = −0.71 and interaction effect B_(cent)_ = −0.18). This represents an approximately 25.4% stronger association in the high-stress group. The full model explained 15% of the variance in SBP (adjusted R^2^ = .15).

**Fig 2:**
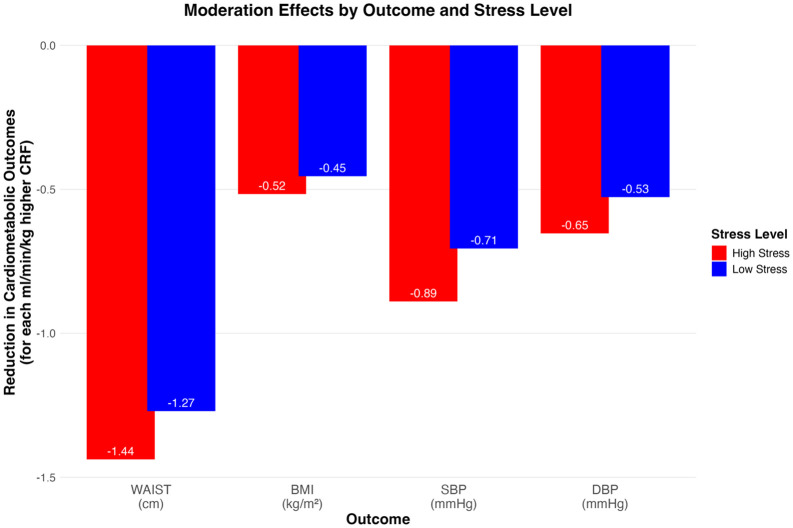
Moderation effect of cardiorespiratory fitness (CRF) on the associations between perceived psychosocial stress and cardiometabolic outcomes. The bars represent the estimated reduction in each outcome per 1 ml/min/kg higher CRF, stratified by low versus high stress.

For each 1 ml/min/kg higher CRF, DBP was decreased by 0.53 mmHg (p < .001) in the low-stress group, with an additional reduction of 0.13 mmHg (p = .020) in the high-stress group, corresponding to a total reduction of 0.65 mmHg for each 1 ml/min/kg higher CRF (combined main effect B_(cent)_ = −0.53 and interaction effect B_(cent)_ = −0.13). This represents an approximately 24.5% stronger association in the high stress-group. The full model explained 12% of the variance in DBP (adjusted R^2^ = .12). Differences in main and total effects across stress-groups are illustrated in Fig 2.

## Discussion

In this large, population-based cohort of middle-aged adults without known disease, higher CRF was found to significantly moderate the adverse associations between psychosocial stress and cardiometabolic risk markers. The buffering effect was evident for waist circumference, BMI, and blood pressure, with the strongest associations among individuals experiencing high levels of stress. These findings highlight CRF as a key modifiable factor mitigating stress-related cardiometabolic dysfunction.

### Associations between CRF, stress and cardiometabolic risk

Participants reporting high stress had lower CRF and more adverse cardiometabolic profiles. These findings align with prior evidence linking higher physical activity levels to lower stress, both cross-sectionally and longitudinally [46], and extend previous findings by demonstrating associations between stress and early markers of cardiometabolic dysregulation, including HDL-C, HbA1c, and glucose. While much of the existing literature focuses on outcomes such as cardiovascular events [25] and mortality [26], our findings highlight associations between stress and subclinical cardiometabolic risk. This supports that there is an association between stress and early symptoms of disease development, prior to overt clinical conditions.

While perceived psychosocial stress showed significant associations with HDL-C in our study, we did not observe significant associations with other lipid markers. These results differ somewhat from previous findings but also extend prior research that has linked stress to a broader cardiometabolic burden. For example, Gerber et al. [7] demonstrated associations between high stress levels and elevated triglycerides, LDL-C, and total cardiometabolic risk, while workplace-related stress research have similarly identified broader lipid dysregulation [48,49].

For instance, in the SCAPIS cohort, Söderberg et al. [50] reported that poor perceived job conditions were significantly associated with low HDL-C (PR = 1.76, CI 95%: 1.25;2.48) as well as diastolic hypertension in women (PR = 1.29, CI: 1.05;1.59), and with increased metabolic syndrome in men (PR = 1.25, CI: 1.02;1.52). And in a large cohort of over 80,000 Swedish women and men from the paid work force, associations have been identified between exposure to stress of workplace sexual harassment and higher incidence of CVDs [51].

The discrepancy between studies may reflect differences in stress measurement approaches, study populations, or the specific stress exposure examined. The use of a single-item measure, while validated for cardiovascular outcomes, for example in the INTERHEART study [25], may capture different dimensions of stress compared to for instance workplace-specific assessments. Additionally, our relatively healthy cohort may not exhibit the full spectrum of stress-related lipid abnormalities that may emerge in higher-risk or clinical populations. While our findings highlight different markers, the observed associations together underscore the role of stress as a contributor to early cardiometabolic dysfunction.

### The moderating role of CRF

We observed that CRF significantly moderated the association between stress and key cardiometabolic risk markers, including waist circumference, BMI, SBP, and DBP. Among individuals reporting high levels of stress, this buffering association was up to 25% stronger than in those experiencing lower stress. These findings suggest that individuals experiencing chronic stress may derive proportionally greater benefits from higher CRF levels, an observation consistent with prior research demonstrating enhanced CRF-related benefits among populations with higher risk profiles [7,13,20,52].

These findings are particularly important given the limited research examining CRF as a moderator of stress-related cardiometabolic outcomes. Most prior studies have investigated self-reported physical activity, which is prone to recall bias or social desirability bias. For example, as early as 1982, Kobasa et al. [35] found that male managers who exercised regularly reported fewer symptoms during periods of high stress. Subsequent studies have supported these observations, showing that individuals who engage in regular physical activity tend to report lower stress levels and fewer physical complaints over time [36]. One longitudinal study of older adults linked habitual aerobic exercise to lower perceived stress and fewer physical complaints over several years of follow-up [53].

Stress-buffering effects from exercise-based interventions have also been demonstrated in clinical settings. In one cardiac rehabilitation study, patients experiencing high psychosocial stress but who improved their exercise capacity had a 60% lower mortality risk than similarly stressed individuals, who did not improve their exercise capacity as much [27].

Although the literature generally supports the stress-buffering role of physical activity, studies focusing explicitly on CRF remain limited and sometimes inconclusive [54,55]. A 2009 systematic review found that while about half of the included studies supported exercise as a stress-buffer across various health outcomes, only a small number examined CRF [36]. Even fewer studies have evaluated CRF in relation to specific cardiometabolic outcomes, among which several report modest or non-significant effects [56].

Consistent with our findings, Gerber et al. [7] observed that individuals with high stress and high CRF had more favourable cardiometabolic profiles, including lower SBP, DBP, LDL-cholesterol, triglycerides, and total metabolic risk, compared to their low-fit counterparts. Notably, unlike our findings, they did not observe a significant interaction with BMI, suggesting that the protective range of CRF may be more extensive than previously identified.

Additionally, laboratory studies have found that individuals with higher CRF show lower physiological reactivity to stress. One study reported a significantly lower increase in SBP (point estimate [PE] = −3.69, CI: −5.27;-2.10) and a trend toward lower DBP (PE = −1.42, CI: −2.90;0.06) in high-fit individuals exposed to laboratory stressors [32]. Our results extend these findings by demonstrating similar effects in a population-based sample, and in relation to long-term rather than acute stress exposure. Beyond cardiometabolic benefits, high CRF has been linked to fewer symptoms of burnout and depression under stress, reinforcing CRF’s broader protective role across multiple health outcomes [57].

### Clinical implications

A 5 cm higher waist circumference has been associated with approximately 9% higher all-cause mortality risk in middle-aged adults [58]. Based on our models, a 5 cm reduction would correspond to an increase in CRF of approximately 3.5–3.9 ml/min/kg, depending on stress level. Structured aerobic training can increase CRF by 15–20% [13], corresponding to gains of approximately 5.1–6.8 ml/min/kg in our cohort (mean 33.9 ml/min/kg), suggesting that commonly achievable CRF improvements could exceed this threshold. Similarly, each 5 kg/m^2^ higher BMI has been associated with 30% and 40% higher all-cause and cardiovascular mortality risk, respectively [59]. These CRF improvements would correspond to BMI reductions of approximately 2.3–3.5 kg/m^2^, potentially sufficient to shift individuals across WHO BMI categories [60]. For blood pressure, even modest 5 mmHg reductions in SBP have been associated with approximately 10% lower risk of major cardiovascular events [61]. The proposed CRF improvements would correspond to reductions of approximately 3.6–6.1 mmHg, approaching this clinically significant threshold, with larger reductions among individuals reporting high stress.

Our findings highlight CRF as a clinically relevant, modifiable factor that may buffer the adverse associations between psychosocial stress and cardiometabolic health, in an apparently healthy middle-aged population. Notably, these buffering associations were strongest in those reporting high stress, suggesting that stressed individuals may derive most benefit from higher CRF levels. Given the high prevalence of individuals experiencing high levels of stress, these findings highlight the potential of CRF as a complementary clinical indicator, when feasible, and underscore the importance of physical activity promotion, especially during periods of high stress. Moreover, individuals with higher CRF showed favourable associations across several risk markers simultaneously, underscoring CRF’s value as a comprehensive marker.

To improve or maintain high CRF, regular aerobic physical activity at moderate intensity or above is required, with current guidelines recommending 150–300 minutes per week of moderate-intensity activity or 75–150 minutes per week of high intensity activity [62]. Individualised approaches may be necessary for individuals experiencing high stress, emphasising graded progression in duration and frequency before advancing intensity [63].

### Strengths and limitations

A major strength of this study is the large, population-based sample and the use of high-quality, standardised measures of both estimated VO_2_max (as an indicator of CRF) and cardiometabolic risk markers. By focusing on CRF rather than self-reported physical activity, we examined a physiological marker which avoids common biases associated with self-report, such as overestimation and recall errors. CRF offers a more objective and integrative indicator of cardiovascular health, incorporating both habitual physical activity and genetic predispositions [42], which consistently has been shown to predict cardiovascular outcomes more reliably than measures of physical activity alone [14].

However, using CRF introduces potential selection bias: about 30% of Gothenburg SCAPIS participants were excluded due to non-participation in CRF testing. Previous analyses have shown that those completing CRF testing are more likely to be women, younger, have lower BMI, higher education, and regular exercise habits [44]. Consequently, our analysed subgroup likely represents a healthier cohort, as individuals with elevated resting heart rates, high body weight, cardiovascular disease, or beta-blocker use were excluded. This limits the generalisability to broader or higher-risk populations. Nonetheless, the observed moderating effects even in this healthier cohort suggest that similar, or even stronger, associations may exist in higher-risk groups.

The cross-sectional study design limits causal inference. Reversed causation represents a key concern: while we interpret higher CRF as buffering stress-related cardiometabolic risk, the observed associations may reflect bidirectional relationships. High stress may impede physical activity, contributing to lower CRF, while simultaneously contributing to adverse cardiometabolic profiles through both behavioural and biological pathways. Conversely, individuals with low CRF may experience greater stress vulnerability. Similarly, individuals with existing cardiometabolic dysfunction may experience elevated stress due to health concerns or functional limitations, while also constraining exercise capacity. Baseline risk differences may also influence both CRF levels and potential for improvement. Furthermore, residual confounding by unmeasured factors such as diet, sleep, or genetic predisposition cannot be excluded. Additionally, the clinical interpretation of our per-unit regression coefficients provided in the Discussion are hypothetical and should be interpreted with caution.

The Ekblom-Bak submaximal test has been validated against direct gas exchange, demonstrating small and non-significant measurement error [45]. Nevertheless, submaximal testing entails inherent uncertainty, partly due to individual variability in heart rate responses, introducing potential errors compared with direct measures. Furthermore, as the test has primarily been validated in Swedish adults, generalisability may be limited. Nevertheless, submaximal testing remains the most feasible method for assessing CRF in large population-based studies and has been consistently associated with cardiovascular morbidity and mortality in the general population, supporting its clinical relevance [11].

Psychosocial stress was measured using a single self-reported item. While practical for large-scale population studies, single-item measures cannot fully capture the intensity, duration, and sources of stress, and is subject to recall bias. Nevertheless, the measure used in this study is well-validated, closely matching the stress item from the INTERHEART study, which has showed strong associations with acute myocardial infarction across 52 countries and diverse populations [25]. Dichotomising stress into “low” and “high” may reduce statistical power, but this approach reflects long-term stress exposure and allows comparison with prior studies [25,46].

Despite these limitations, our findings support a stress-buffering role of CRF, especially under high stress, regardless of the specific mechanisms through which this protection operates. Future longitudinal studies are needed to explore dose–response relationships and establish causality.

## Conclusions

In this population-based cohort of middle-aged adults without known disease, CRF significantly attenuated the adverse associations between psychosocial stress and key cardiometabolic risk markers, including waist circumference, BMI, and blood pressure. The associations were particularly pronounced among individuals with high stress, suggesting that CRF may buffer stress-related cardiometabolic deterioration. Our findings add to the growing body of evidence suggesting that during periods of high stress, engaging in physical activity, which is the major determinant of CRF, may be an effective strategy to prevent stress-related cardiometabolic decline. Given generally low physical activity levels in the population [64], the moderating potential of CRF suggests that public health prevention strategies could benefit from targeting improved physical activity levels in the population.
